# PET imaging of mitochondrial complex-I in the adenine-induced tubulointerstitial nephropathy mouse model using [^18^F]BCPP-BF

**DOI:** 10.1186/s41181-025-00392-1

**Published:** 2025-10-13

**Authors:** Kenneth Dahl, Peter Johnström, Miklós Toth, Vasco C. Sousa, Charlotte Ericsson, Maria Strömstedt, Tord Inghardt, Miguel A. Cortés González, Anna Reznichenko, Aurelija Jucaite, Zsolt Cselényi, Robert Unwin, Hiroyuki Ohba, Christer Halldin, Benjamin Challis, Hideo Tsukada, Magnus Schou

**Affiliations:** 1 PET Science Centre, Precision Medicine and Biosamples, Oncology R&D, AstraZeneca, Karolinska Institutet, Stockholm, Sweden; 2 Department of Clinical Neuroscience, Centre for Psychiatry Research, Karolinska Institutet and Stockholm County Council, Stockholm, Sweden; 3 Division of Imaging Core Facilities, Department of Clinical Neuroscience, Center for Imaging Research, Karolinska Institutet, Stockholm, Sweden; 4 Bioscience, Early Cardiovascular, Renal and Metabolism, Biopharma R&D, AstraZeneca, Gothenburg, Sweden; 5 Chemistry, Early Cardiovascular, Renal and Metabolism, Biopharma R&D, AstraZeneca, Gothenburg, Sweden; 6 Translational Science and Experimental Medicine, Early Cardiovascular, Renal and Metabolism, Biopharma R&D, AstraZeneca, Gothenburg, Sweden; 7 Early Clinical Development, Early Cardiovascular, Renal and Metabolism, Biopharma R&D, AstraZeneca, Cambridge, UK; 8 Central Research Laboratory, Hamamatsu Photonics K. K., Hamamatsu, Japan; 9 Department of Biophysics and Radiation Biology, HUN-REN TKI, Semmelweis University, Budapest, Hungary

**Keywords:** Positron emission tomography, MC-I, Radiopharmaceutical, Kidney disease

## Abstract

**Background:**

Chronic kidney disease (CKD) poses a significant global health burden with limited effective treatments for its prevention, progression, and associated complications. Mitochondrial dysfunction is recognized as a pivotal factor in the development of kidney diseases, with mitochondrial complex-I (MC-I) playing a crucial role in assessing overall mitochondrial function. Recent advancements in selective MC-I positron emission tomography (PET) radioligands now allow for non-invasive visualization and quantification of renal mitochondrial status in vivo. The aim of the present study was to evaluate the utility of [^18^F]BCPP-BF in the adenine induced tubulointerstitial nephropathy model.

**Results:**

Binding of the MC-I targeted PET radioligand, [^18^F]BCPP-BF, showed a gradual decline in the kidneys of mice on an adenine-rich diet. [^18^F]BCPP-BF binding decreased by 59–61% compared to baseline after two weeks of adenine treatment. These results of reduced uptake were further confirmed by in vitro autoradiography. In kidneys from adenine-fed mice, [^18^F]BCPP-BF specific binding was reduced by 65.6% compared to control kidney sections.

**Conclusions:**

Altogether, the findings suggest that [^18^F]BCPP-BF holds potential as an imaging biomarker for renal failure. However, further preclinical studies and validation in human subjects are necessary before it can be established as a reliable indicator for the progression of CKD.

**Supplementary Information:**

The online version contains supplementary material available at 10.1186/s41181-025-00392-1.

## Introduction

Chronic kidney disease (CKD) is an irreversible and progressive condition that is increasing dramatically in the global population (Xie et al. 2018; Romagnani et al. 2017;). Given that current treatments can only slow down the progression of the disease and prevent some related complications, early detection and diagnosis of renal impairment is crucial for the prognosis of those affected to facilitate early therapeutic intervention/treatment.

The kidney is a highly energy-demanding and mitochondria-rich organ. It is believed that mitochondrial dysfunction plays a critical role in the onset and advancement of acute and chronic kidney diseases and is an important target for potential new therapies (Zhang et al. 2021; Che et al. 2014). Mitochondrial complex I (MC-I) is the first enzyme in the respiratory chain and is a vital component of bioenergetics, making it a potential biomarker for overall mitochondrial function.

Positron emission tomography (PET) is a molecular imaging technique used to study the distribution of radiolabeled tracer molecules (radioligands) in vivo in a non-invasive fashion (Ametamey et al. 2008; Miller et al. 2008). MC-I PET radioligands have demonstrated significant promise across multiple disease areas, and in particularly within neurodegenerative disorders (e.g., Alzheimer’s disease) and cardiovascular research (Terada et al. 2021; Yu et al. 2007). More recently, [^18^F]BCPP-BF, a novel MC-I antagonist, has been utilized as a specific PET radioligand to visualize and quantitate the progressive reduction of renal MC-I in four different preclinical models of kidney injury (Ohba et al. 2016; Saeki et al. 2020; Srivastava et al. 2020; Sato et al. 2025). Further to this, a recent first-in-human study thoroughly evaluated and confirmed the safety and in vivo biodistribution of [^18^F]BCPP-BF (Sato et al. 2025). The aim of the present study was to evaluate the utility of [^18^F]BCPP-BF in the adenine induced tubulointerstitial nephropathy model (Jia et al. 2013).

## Materials and methods

Unless otherwise stated all reagents were obtained from commercially available sources and used without further purification. The precursor and the reference standard for BCPP-BF were provided by Hamamatsu photonics (Hamamatsu, Japan).

### Radiochemistry

Radioligand [^18^F]BCPP-BF was synthesized using similar conditions as previously described (Harada et al. 2013). The in housed developed synthesis protocol for [^18^F]BCPP-BF was performed using a semi-automated ^18^F-synthesis platform (DMAutomation, Sweden). In brief, fluorine-18 was obtained in the chemical form of [^18^F]fluoride ion ([^18^F]F^−^) via the ^18^O(p, n)^18^F nuclear reaction, by irradiating a cyclotron-target containing [^18^O]water with a proton beam (16.4 MeV) of 35 µA for 5–8 min (PETtrace 800 cyclotron, GE, Uppsala, Sweden). The typical amount of [^18^F]fluoride produced in the cyclotron target was approximately 10 GBq (270 mCi). [^18^F]F^−^ was first trapped on an Oasis WAX (1 cc, 30 mg, Waters) anion exchange cartridge and subsequently eluted from the cartridge with a solution of Et_4_NHCO_3_ (10.5 µmol, 2.0 mg) in water (42 µL, 18 MΩ), and CH_3_CN (958 µL) to a reaction vessel (10 mL). The solvents were evaporated at 110 °C for 10 min under continuous nitrogen flow (100 mL/min) to form a dry complex of [^18^F]tetraethylammonium fluoride ([^18^F]TEAF). 7 mg of BCPP-BF precursor in 1.0 mL of CH_3_CN was added and the resulting solution was heated to 80 °C for 10 min. The crude reaction mixture was cooled and diluted with 1.0 mL water, after which [^18^F]BCPP-BF was injected onto a semi-preparative HPLC column (ACE-5, C18-HL, 10 × 250 mm) for purification (Fig. S5 (supporting information)). The column outlet was connected to a UV absorbance detector (λ = 290 nm) in series with a GM tube for radioactivity detection. HPLC separation was performed using a mobile phase consisting of CH₃CN/H₂O (60/40) at a flow rate of 6.0 mL/min.The desired radioactive fraction (Rt = 15 min) was diluted with sterile water (50 mL). The resulting mixture was loaded onto a preconditioned (10 mL of ethanol followed by 10 mL of sterile water) SepPak Vac cartridge (1 cc, tC18, Waters). The cartridge was washed with sterile water (10 mL), and [^18^F]BCPP-BF was finally eluted with 0.8 mL of ethanol. The product was finally formulated in 8 mL of Saline (0.9% NaCl, pH 4.5–7.0, ApoEx, Sweden) containing 2% Tween 80 (Sigma-Aldrich) and sterile filtrated by passing through a 0.22-µm sterile Millex-GV filter (Merck KGaA, Germany). [^18^F]BCPP-BF was obtained in an overall radiochemical yield (RCY) of 30–40% (non-decay-corrected). Radiochemical purity (RCP) and molar activity (Am) were >99% and 59 ± 48 GBq/µmol, respectively, with a radiosynthesis and purification time of 70 min. The RCP and Am was determined by analytical radio-HPLC (Fig. S6 (supporting information)).

### The adenine kidney mice model and study design

Male C57BL/6NCrl mice, aged 7 weeks were purchased from SCANBUR A/S (Karlslunde, Denmark). For the in vivo study, a total of 18 mice were included in the study and all were given the adenine diet (0.2% adenine; OpenStandard Diet D11112201) for 14 days. PET scans were performed at baseline (day 0), after 7 days and 14 days. On the PET experiment days blood sampling was taken for analysis of biomarkers. Also, on day 7 a subset (*n* = 4) of the animals was sacrificed for organ harvesting and tissue analysis. Following the last PET measurement on day 14 the remaining animals were sacrificed, and organs harvested.

For the in vitro autoradiography, a total of 15 mice were included in the study, of which 9 mice were given the adenine diet (0.2% adenine; OpenStandard Diet D11112201) for 14 days, while 6 mice were on a normal (control) diet. After 14 days all of the animals were sacrificed for tissue analysis and blood serum analysis. The kidneys were snap frozen and kept at -80 °C.

All mice were housed under the same conditions with access to food and water ad libitum and a 12-h light/dark cycle. All experimental procedures complied with the guidelines and regulations of the Swedish National Board for Laboratory Animals, and the Regional Ethics and Animal Research Committee at Karolinska Institutet approved the study protocol (Dnr.: 4777-2021).

### In vitro autoradiography

Sectioning of the kidney tissue and autoradiography assays were performed at the Autoradiography Core Facility, Department of Clinical Neuroscience, Division of Imaging Core Facilities and Center for Imaging Research (CIR), at the Karolinska Institutet.

Snap-frozen kidney blocks were sectioned at 10 μm thickness using a cryomicrotome (CM1860, Leica Biosystems, Nußloch, Germany), thaw-mounted onto glass slides and stored at -20 °C until the autoradiography assay was performed.

For the [^18^F]BCPP-BF binding autoradiography assay, all sections were first pre-incubated for 20 min at room temperature with binding buffer (50 mM Tris HCl, pH 7.4, containing 120mM NaCl, 5mM KCl, 2mM CaCl2, 1mM MgCl2), then incubated at room temperature with [^18^F]BCPP-BF (0.1 MBq/mL, 31.2 GBq/µmol) for 55 min. Non-specific binding was determined by co-incubation with 10 µM of unlabelled BCPP-BF or 10 µM Rotenone (Merck KGaA, Darmstadt, Germany). After incubation, sections were washed 3 × 5 min at 4 °C, in 50 mM Tris HCl, pH 7.4, briefly dipped in ice-cold distilled water, then dried in a heat plate before being exposed to storage phosphor screens (BAS-IP SR2025, Fujifilm, Tokyo, Japan) overnight.

Radioactivity was detected and quantified with an Amersham Typhoon FLA-9500 phosphor imaging scanner (Cytiva, Marlborough, MA, USA). Autoradiograms were analyzed using Multi Gauge 3.2 phosphor imager software (Fujifilm, Tokyo, Japan). The measured photo-stimulated luminescence (PSL)/mm2 values were converted into decay-corrected radioactivity units based on the signal intensity from standard quantities (4 Bq to 484 Bq), diluted from the [^18^F]BCPP-BF batch, and pipetted (20 µL per standard) onto filter papers exposed in each storage phosphor plate. Binding values from four replicates of total and non-specific conditions from each individual were plotted using GraphPad Prism v10 (GraphPad Software, Boston, MA, USA) and specific binding was determined by subtracting the binding signal in the presence of 10 µM rotenone from the total binding.

### PET imaging in mice

**Fig. 1:**
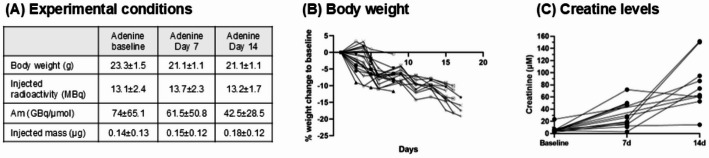
Adenine model development. **A** Experimental conditions **B** Body weight until termination. **C** Creatine levels at baseline and at 7 and 14 days into the study

All PET measurements were performed using the Mediso nanoScan^®^ PET/MRI (1 Tesla MRI system) and the nanoScan^®^ PET/CT small animal imaging systems. Two animals were examined at the same time (one mouse in each PET camera) in the identical PET modules of the two imaging systems that had previously been cross calibrated with each other. On the experimental day, the animal was anesthetized with inhalation of 4–5% of isoflurane. After induction of anesthesia, the isoflurane concentration was lowered to 1.5–2%, and the animal was placed in the scanner. A 93-min dynamic PET measurement was initiated immediately on intravenous injection of [^18^F]BCPP-BF. The image reconstruction was made with a fully 3-dimensional penalized maximum-likelihood algorithm (MLEM; Tera-Tomo; Mediso Ltd.) with 10 iterations, 6 subsets and 0.4 mm voxel size (without scatter or attenuation correction). Data on the weight of the animals, injected radioactivity levels and injected mass at each time point are displayed in Fig. 1A.

### PET image analysis

Each animal’s dynamic PET image was analyzed in PMOD (PMOD Technologies Ltd., Zurich). Decay corrected time activity curves (TAC) were generated using the volume of interest (VOI) delineated in PMOD with the help of MRI images (20 min T1 Spinecho 2D Coronal) collected the same day. To normalize for injected activity and body weight the regional radioactivity values were expressed as standard uptake values (SUV = regional activity * body weight / injected radioactivity). Area under the curve (AUC) values for the TAC was analyzed using GraphPad Prism 9.

## Results

### Establishment of the adenine mice model

As expected, a gradual weight loss was observed in mice fed with the adenine diet (Fig. 1A, B) (Jia et al. 2013). Other than some weight loss, no serious adverse events were observed, and no animals were lost during the study. Also, consistent with results reported by Jia et al., a gradual increase in serum creatinine levels was observed in adenine-fed animals (Figs. 1C and 3S (supporting information)).

### [^18^F]BCPP-BF binding is gradually reduced in the kidneys of adenine-fed mice

**Fig. 2:**
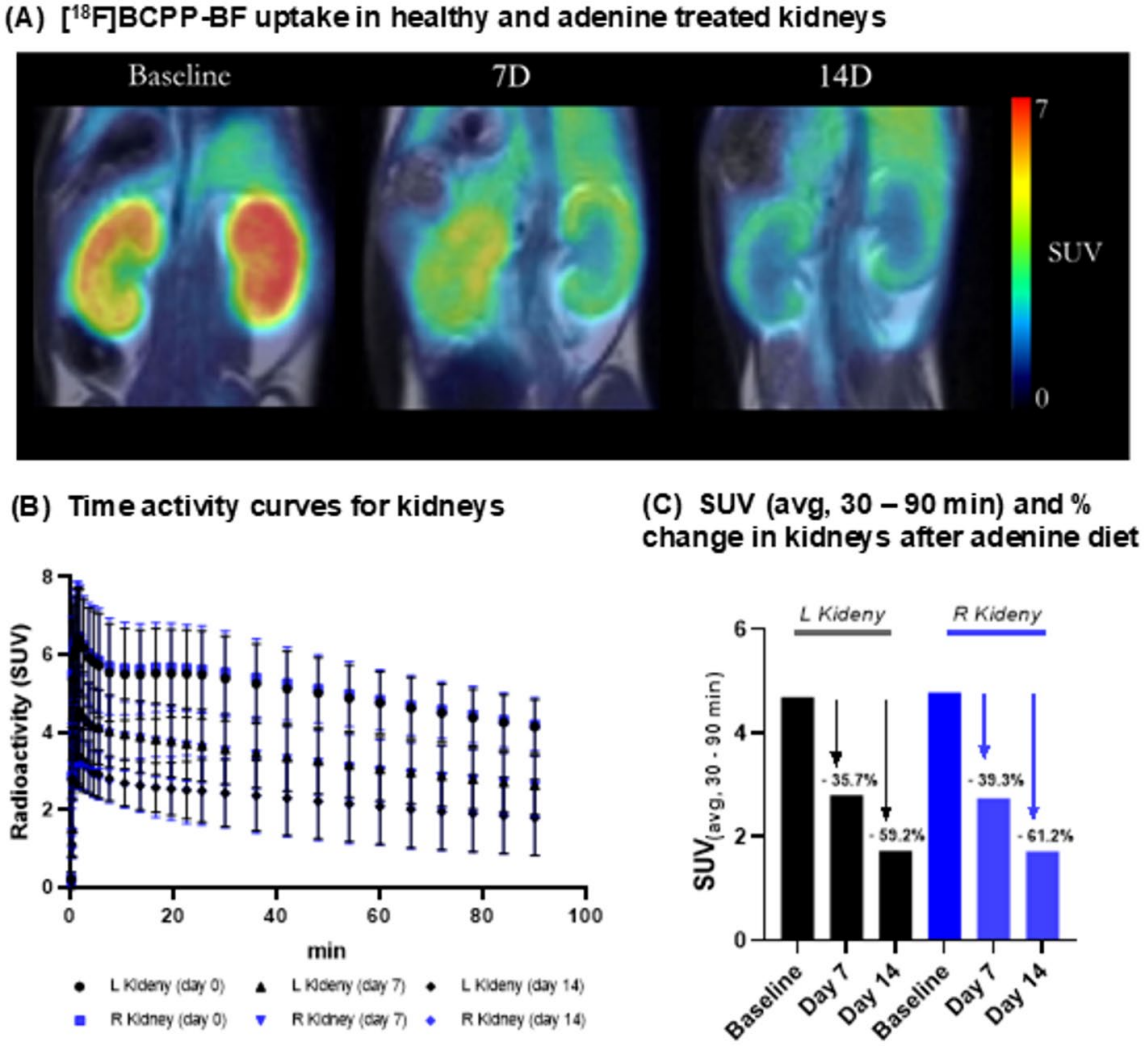
Radioactivity in kidney following the intravenous injection of [^18^F]BCPP-BF in a mice model of adenine-induced renal failure. **A** Summation PET images (3–93 min) at baseline (day 0, left), day 7 (middle), and day 14 (right). **B** Time activity curves (TACs) generated for the left and right kidney. **C** Bar grapgh summarizing the standardized uptake values (SUV_avg_, 30–90 min) for the left and right kidneys at baseline and relative change (%) after adenine diet

Whole-body dynamic PET measurements were performed at days 0 (baseline), 7 and 14 after initiation of the adenine diet. Following injection of [^18^F]BCPP-BF, radioactivity was rapidly distributed to the kidneys, where it was preferentially distributed to the renal cortex (Fig. 2A). Kidney time-activity curves (TACs) peaked at 10–15 min after radioligand injection and were followed by a slow wash-out (Fig. 2B). The standardized uptake value (SUV_avg_, 30–90 min post radioligand injection) of [^18^F]BCPP-BF in kidneys at baseline ranged from 4.7 to 4.8 SUV_avg_, which dropped to 2.7–2.8 SUV_avg_ after 7 days of adenine diet, and to 1.7 SUV after 14 days of adenine diet (Fig. 2C). The percentage reduction in [^18^F]BCPP-BF binding relative to baseline corresponded to 36–39% (day 7, Fig. 2C) after the first week of treatment and a further decrease to 59–61% at the end of the study (day 14, Fig. 2C). A comparable reduction in [^18^F]BCPP-BF uptake was also observed when comparing the area under the curve (AUC, 0–90 min) for treated mice compared to baseline. As shown in Fig. 1S (supporting information), uptake decreased by 33–36% on day 7 and 54–56% on day 14.

### In vitro characterization of [^18^F]BCPP-BF binding in mice kideny tissue

**Fig. 3:**
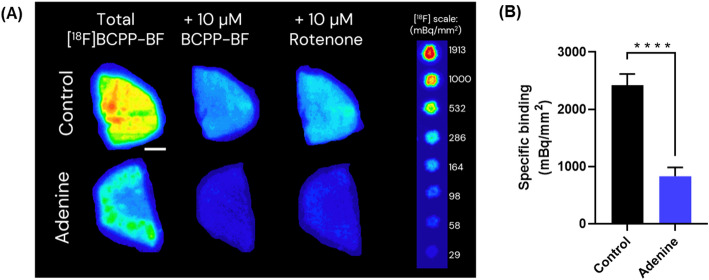
[^18^F]BCPP-BF binding autoradiography in kidney tissue sections from Control mice and mice fed with adenine in their diet for 14 days (Adenine). **A** Representative autoradiograms of [^18^F]BCPP-BF (0.1 MBq/mL, 31.2 GBq/µmol) binding alone (Total) and co-incubated with excess amount (10 µM) of unlabelled BCPP-BF or Rotenone in kidney from *n* = 1 control and *n* = 1 adenine-fed mice. Line scale: 1 mm. The fluorine-18 standard scale shows the decay-corrected values used for density calibration. **B** Specific [^18^F]BCPP-BF Binding (mBq/mm^2^), calculated by subtracting the signal obtained co-incubation of the tracer with 10 µM Rotenone from the Total signal. Bars represent the mean ± s.e.m. of *n* = 6 control and *n* = 9 adenine-fed mice. Each measurement is the average from 4 replicate sections. **** *p* < 0.0001, two-tailed unpaired t-test

**Fig. 4:**
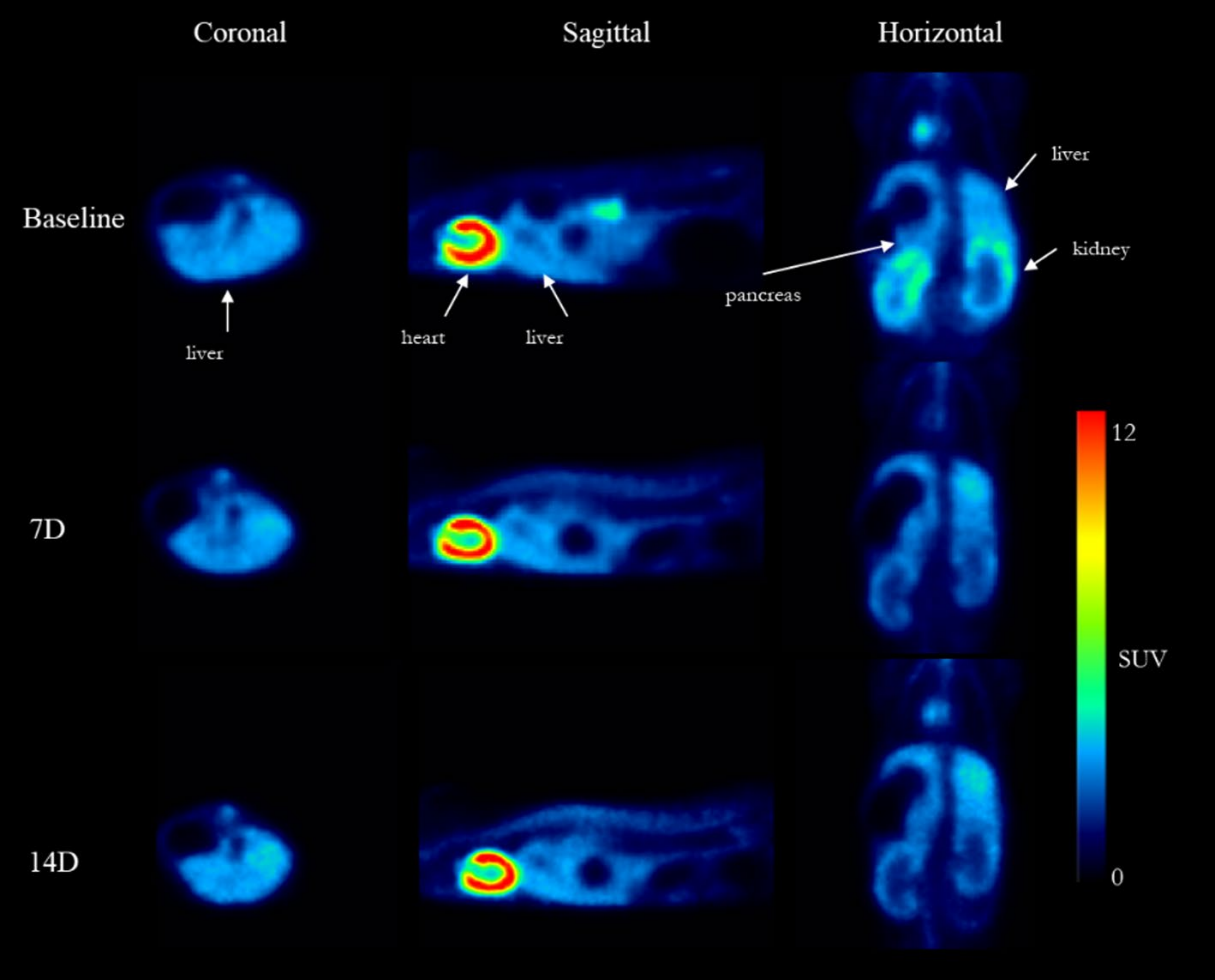
Radioactivity in the heart/left ventricle, liver, and pancreas following the intravenous injection of [^18^F]BCPP-BF in a mice model of adenine-induced renal failure. Summation PET images (3–93 min) at baseline (day 0, top), day 7 (middle), and day 14 (bottom)

[^18^F]BCPP-BF binding was evaluated by autoradiography in kidney sections of *n* = 6 control and *n* = 9 adenine-fed mice (Fig. 3). [^18^F]BCPP-BF binding was strongly specific, as the average total signal in control mouse kidney (2.9 Bq/mm^2^) was nearly fully displaced with 10 µM unlabelled BCPP-BF (85.8 ± 1.1%) and Rotenone (83.6 ± 1.7%) (Fig. 4S (supporting information)). Taking the binding displaced by rotenone as the specific binding signal (Fig. 3B), [^18^F]BCPP-BF specific binding in adenine-fed mouse kidneys was 65.6 ± 2.1% lower, compared to control kidney sections (*p* < 0.0001, two-tailed unpaired t-test).

### [^18^F]BCPP-BF binding in other organs

The distribution of [^18^F]BCPP-BF to heart/left ventricle, liver, and pancreas was also investigated. The highest radioactivity was observed in the whole heart (10.5 SUV_avg_, 33–93 min post radioligand injection), followed by the left ventricle (3.5 SUV_avg_), liver (2.7 SUV_avg_), and pancreas (2.1 SUV_avg_). Representative summation PET images are shown in Fig. 4. As observed in the kidney, the initial peak in organ TACs was followed by a slow washout (Fig. 2S, supporting information). A significantly higher accumulation of radioactivity was observed in the liver following [^18^F]BCPP-BF administration, particularly at the early time points (0–33 min) after 2 weeks of adenine treatment compared to baseline SUV values (see Tables S1 and S2, supporting information). Only non-significant changes were observed in the TACs for the other organs imaged.

## Discussion

Several animal models of chronic kidney disease (CKD) have been developed to study effects of novel drug treatments, both using surgical (e.g., 5/6 nephrectomy) and chemical (e.g., kidney ischemia-reperfusion) interventions. However, most animal models do not mimic the complexity of human disease. The non-surgical adenine diet model of CKD in rodents (Jia et al. 2013) has gained a lot of attention in recent years, because it is known to produce a model that resembles the slow progression of CKD in humans.

In this study, the PET radioligand [^18^F]BCPP-BF was utilized to investigate MC-I in the adenine-induced mouse model. Although this radioligand has previously been applied in preclinical PET studies to visualize MC-I in animal models of renal failure (Ohba et al. 2016; Saeki et al. 2020), to the best of our knowledge this is the first evaluation in vivo of MC-I radioligand binding in a widely-used mouse model of CKD.

An advantage with PET is its non-invasive nature, which in this study enabled us to follow the adenine-induced decrease in MC-I binding over time without the need for a control group. Indeed, a substantial reduction in the PET signal was observed during the study, nearly 40% after the first week and 60% after two weeks. To further corroborate these in vivo findings, it was confirmed that [^18^F]BCPP-BF exhibits high specificity in binding to MC-I in vitro. Similarly, as observed in our in vivo study, mice on an adenine-rich diet showed a significant 65% reduction in radioligand binding compared to those on a control diet. This decrease was associated with an increase in serum creatinine levels, thereby confirming that this reduction was accompanied by reduced kidney function. Further studies is however necessary to better understand the impact of blood flow on [^18^F]BCPP-BF uptake in the kidneys of mice treated with adenine. It remains possible that the observed reduction in [^18^F]BCPP-BF uptake in vivo may partially be attributed to decreased blood perfusion in the kidneys due to adenine treatment. However, such functional studies, e.g., prefusion MRI (Gutjahr et al. 2017), were outside the scope of this study. These preliminary results, along with our earlier studies using [^18^F]BCPP-BF in other kidney injury models (Ohba et al. 2016; Saeki et al. 2020), further highlights the potential of [^18^F]BCPP-BF as an imaging biomarker for renal failure.

PET radioligands targeting MC-I were first developed as sensitive biomarkers to assess cardiac function (Kim et al. 2018; Yu et al. 2007; Marshall et al. 2004). Thus, the distribution of [^18^F]BCPP-BF to other organs, including the heart, was also investigated in the adenine mouse model. As expected, the uptake of [^18^F]BCPP-BF into the rodent heart was substantial (10.5 SUV_avg_); however, no significant differences were observed following the two weeks treatment period. The only effects of adenine treatment, apart from in the kidneys, were observed in the liver. Significant differences were observed in the early and late time frames of the activity curves. More studies are needed to confirm these preliminary findings.

The relatively small sample size is a limiting factor of the present study. Another limitation is that, in the absence of arterial blood sampling, no quantification of radioligand binding was performed. Although the results are consistent within the groups and between animals, further preclinical and clinical studies are needed to validate [^18^F]BCPP-BF and MC-I PET as a valuable quantitative imaging approach, and as a potential biomarker both for drug development in CKD and monitoring of disease course progression and for drug development in CKD.

## Conclusions

A progressive and gradual decline in the [^18^F]BCPP-BF PET signal was observed in the adenine-induced tubulointerstitial nephropathy mouse model. These findings of reduced uptake were corroborated by in vitro binding data using [^18^F]BCPP-BF. Overall, these preliminary results underscore the potential of [^18^F]BCPP-BF as a candidate imaging biomarker for renal failure. Nonetheless, additional preclinical studies and validation in human subjects are necessary before [^18^F]BCPP-BF can be endorsed as a reliable biomarker for the progression of chronic kidney disease.

## Supplementary Information

Below is the link to the electronic supplementary material.

Supplementary Material 1

## Data Availability

Data will be made available on reasonable request.

## References

[CR1] Ametamey SM, Honer M, Schubiger PA. Molecular imaging with PET. Chem Rev. 2008;108(5):1501–16. 18426240 10.1021/cr0782426

[CR2] Che R, Yuan Y, Huang S, Zhang A. Mitochondrial dysfunction in the pathophysiology of renal diseases. Am J Physiol Ren Physiol. 2014;306:367–78. 10.1152/ajprenal.00571.2013 24305473

[CR3] Gutjahr FT, Günster SM, Kampf T, Winter P, Herold V, Bauer WR, Jakob PM. MRI-based quantification of renal perfusion in mice: improving sensitivity and stability in FAIR ASL. Z Med Phys. 2017;27:334–39. 28431859 10.1016/j.zemedi.2017.02.001

[CR4] Harada N, Nishiyama S, Kanazawa M, Tsukada H. Development of novel PET probes, [^18^F]BCPP-EF, [^18^F]BCPP-BF, and [^11^C]BCPP-EM for mitochondrial complex 1 imaging in the living brain. J Label Compd Radiopharm. 2013;56:553–61. 10.1002/jlcr.3056 24285187

[CR5] Jia T, Olauson H, Lindberg K, Amin R, Edvardsson K, Lindholm B, Andersson G, Wernerson A, Sabbagh Y, Schiavi S, Larsson TE. A novel model of adenine-induced tubulointerstitial nephropathy in mice. BMC Nephrol. 2013;14:116–24. 23718816 10.1186/1471-2369-14-116 PMC3682934

[CR6] Kim D, Cho S, Bom H. Emerging tracers for nuclear cardiac PET imaging. Nucl Med Mol Imaging. 2018;52:266–78. 30100939 10.1007/s13139-018-0521-1 PMC6066491

[CR7] Marshall RC, Powers-Risius P, Reutter BW, O’Neil JP, La Belle M, Huuesman RH, et al. Kinetic analysis of ^18^F-Fluorodihydrorotenone as a deposited myocardial flow tracer: comparison to ^201^Tl. J Nucl Med. 2004;45(11):1959–59. 15534068

[CR8] Miller PW, Long NJ, Vilar R, Gee AD. Synthesis of ^11^C, ^18^F, ^15^O, and ^13^N radiolabels for positron emission tomography. Angew Chem Int Ed. 2008;47:8998–9033. 10.1002/anie.200800222 18988199

[CR9] Ohba H, Kanazawa M, Kakiuchi T, Tsukada H. Effects of acetaminophen on mitochondrial complex I activity in the rat liver and kidney: a PET study with ^18^F-BCPP-BF. EJNMMI Res. 2016;6:82. 27873239 10.1186/s13550-016-0241-4 PMC5118230

[CR10] Romagnani P, Remuzzi G, Glassock R, Levin A, Jager KJ, Tonelli M, Massy Z, Wanner C, Anders H-J. Chronic kidney disease. Nat Rev. 2017;3:17088. 10.1038/nrdp.2017.88 29168475

[CR11] Saeki S, Ohba H, Ube Y, Tanaka K, Haruyama W, Uchii M, Kitayama T, Tsukada H, Shimada T. Positron emission tomography imaging of renal mitochondria is a powerful tool in the study of acute and progressive kidney disease models. Kidney Int. 2020;98(1):88–99. 32471638 10.1016/j.kint.2020.02.024

[CR12] Sato K, Bunai T, Oda A, Ohba H, Sano Y, Tokui A, Tsukada H, Nishizawa S, Harada N, Ouchi Y. Biodistribution of [^18^F]BCPP-BF in humans: a first-in-human positron emission tomography study. Nucl Med Biol. 2025;144–145:109019. 40250068 10.1016/j.nucmedbio.2025.109019

[CR13] Srivastava A, Prasad PV. Visualizing mitochondrial (dys) function using positron emission tomography imaging. Kidney Int. 2020;98:51–3. 32571489 10.1016/j.kint.2020.03.021 PMC7745541

[CR14] Terada T, Therriault J, Kang MSP, Savard M, Pascoal TA, Lussier F, Tissot C, Wang Y-T, Benedet A, Matsudaira T, Bunai T, Obi T, Tsukada H, Ouchi Y, Rosa-Neto P. Mitochondrial complex I abnormalities is associated with Tau and clinical symptoms in mild Alzheimer’s disease. Mol Neurodegener. 2021;16:28. 33902654 10.1186/s13024-021-00448-1 PMC8074456

[CR15] Xie Y, Bowe B, Mokdad AH, Xian H, Yan Y, Li T, Maddukuri G, Tsai C, Floyd T, Al-Aly Z. Analysis of the global burden of disease study highlights the dlobal, reginal, and national trends of chronic kidney disease epidemiology from 1990 to 2016. Kidney Int. 2018;94:567–81. 30078514 10.1016/j.kint.2018.04.011

[CR16] Yu M, Guaraldi MT, Mistry M, Kagan M, McDonald JL, Drew K, Radeke H, Azure M, Purohit A, Casebier DS, Robinson SP. BMS-747 158-02: a novel PET myocardial perfusion imaging agent. J Nucl Cardiol. 2007;14(6):789–98. 18022105 10.1016/j.nuclcard.2007.07.008

[CR17] Zhang X, Agborbesong E, Li X. The role of mitochondria in acute kidney injury and chronic kidney disease and its therapeutic potential. Int J Mol Sci. 2021;22(20):11253–75. 34681922 10.3390/ijms222011253 PMC8537003

