# Supplementary material for: PET imaging of mitochondrial complex-I in the adenine-induced tubulointerstitial nephropathy mouse model using [18F]BCPP-BF
Source: EJNMMI Radiopharm Chem. 2025 Oct 13;10:66. doi: 10.1186/s41181-025-00392-1 (PMC12518723; doi:10.1186/s41181-025-00392-1)
Supplement: Supplementary file 1 — Supplementary Material 1 [file 41181_2025_392_MOESM1_ESM.docx]

**PET imaging of mitochondrial complex-I in the adenine-induced tubulointerstitial nephropathy mouse model using [^18^F]BCPP-BF**

Kenneth Dahl^1,2,^*, Peter Johnström^1,2^, Miklós Toth^2^, Vasco C. Sousa^3^, Charlotte Ericsson^4^, Maria Strömstedt^4^, Tord Inghardt^5^, Miguel Cortes Gonzalez^2^, Anna Reznichenko^6^, Aurelija Jucaite^1,2^, Zsolt Cselényi^1,2^, Robert Unwin^7^, Hiroyuki Ohba^8^, Christer Halldin^2,9^, Benjamin Challis^5^, Hideo Tsukada^7^, Magnus Schou^1,2^

^1^PET Science Centre, Precision Medicine and Biosamples, Oncology R&D, AstraZeneca, Karolinska Institutet, Stockholm, Sweden.

^2^Department of Clinical Neuroscience, Centre for Psychiatry Research, Karolinska Institutet and Stockholm County Council, Stockholm, Sweden

^3^Department of Clinical Neuroscience, Division of Imaging Core Facilities, Center for Imaging Research, Karolinska Institutet, Stockholm, Sweden.

^4^Bioscience, Early Cardiovascular, Renal and Metabolism, Biopharma R&D, AstraZeneca, Gothenburg, Sweden

^5^Chemistry, Early Cardiovascular, Renal and Metabolism, Biopharma R&D, AstraZeneca, Gothenburg, Sweden

^6^Translational Science and Experimental Medicine, Early Cardiovascular, Renal and Metabolism, Biopharma R&D, AstraZeneca, Gothenburg, Sweden

^7^Early Clinical Development, Early Cardiovascular, Renal and Metabolism, Biopharma R&D, AstraZeneca, Cambridge, United Kingdom

^8^Central Research Laboratory, Hamamatsu Photonics K. K., Hamamatsu, Japan

^9^HUN-REN TKI, Department of Biophysics and Radiation Biology, Semmelweis University, Budapest, Hungary

**Supporting Information**

**Content**

1. [^18^F]BCPP-BF binding data in kidneys
2. [^18^F]BCPP-BF binding data in heart, left ventricle, liver, and pancreas.
3. In vitro autoradiography data with [^18^F]BCPP-BF
4. [^18^F]BCPP-BF semipreparative and analytical chromatograms.
5. **[^18^F]BCPP-BF binding data in kidneys**

**
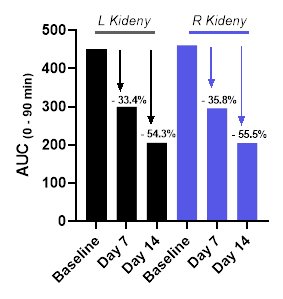
**

**Fig S1.** Radioactivity in kidney following the intravenous injection of [^18^F]BCPP-BF in a mice model of adenine-induced renal failure. Plot summarizing the area under the curve (AUC, 0 – 90 min) for the kidneys at baseline and relative change (%) after adenine diet.

1. **[^18^F]BCPP-BF binding data in heart, left ventricle, liver, and pancreas**


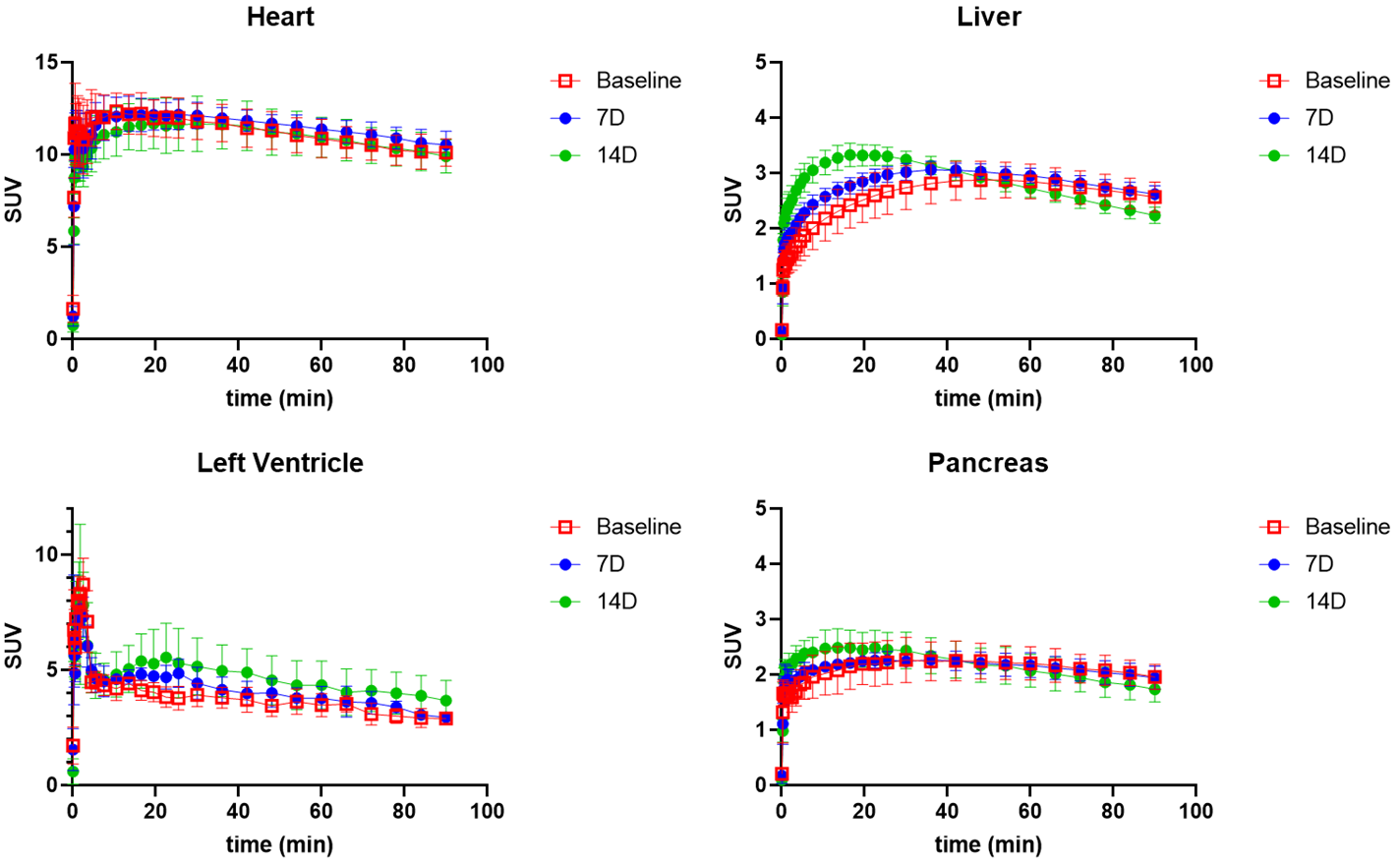


**Fig S2.** Radioactivity in the heart, left ventricle, liver, and pancreas following the intravenous injection of [^18^F]BCPP-BF in a mice model of renal failure.


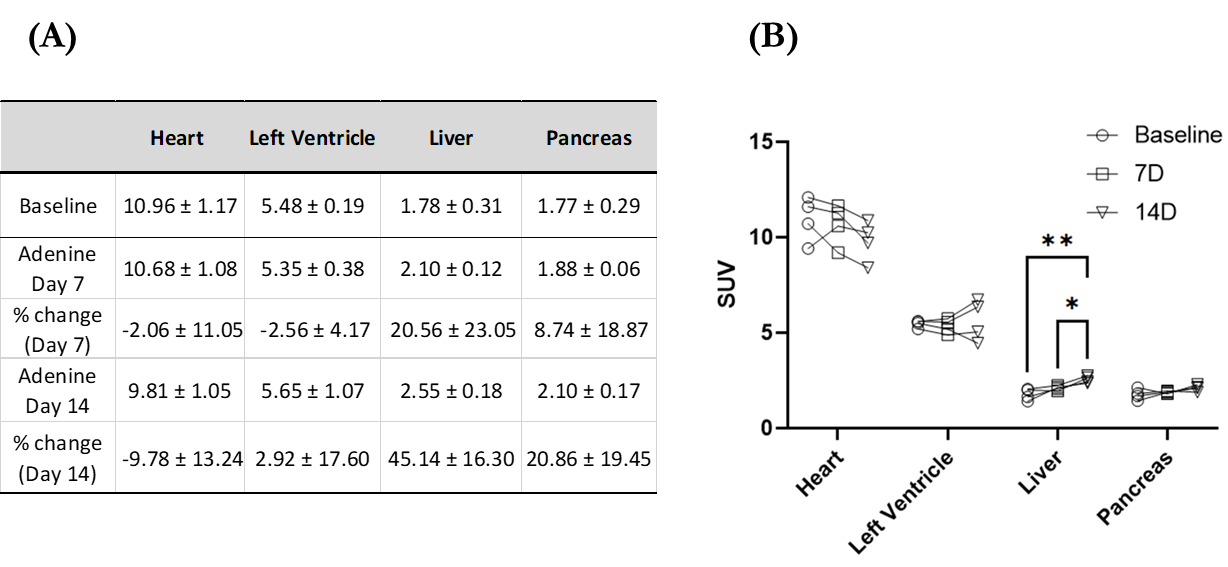


**Table S1.** Early (0 – 33 min) SUV values in the heart, left ventricle, liver, and pancreas following the intravenous injection of [^18^F]BCPP-BF. Average of early SUV values (n = 4) at baseline, Day 7, and Day 14. The data also include % change compared to baseline (A). Same SUV data as in (A) but displayed in a plot format (B). 2-way ANOVA, with Tukey's multiple comparison (*p=0.041; **p=0.0031).

**
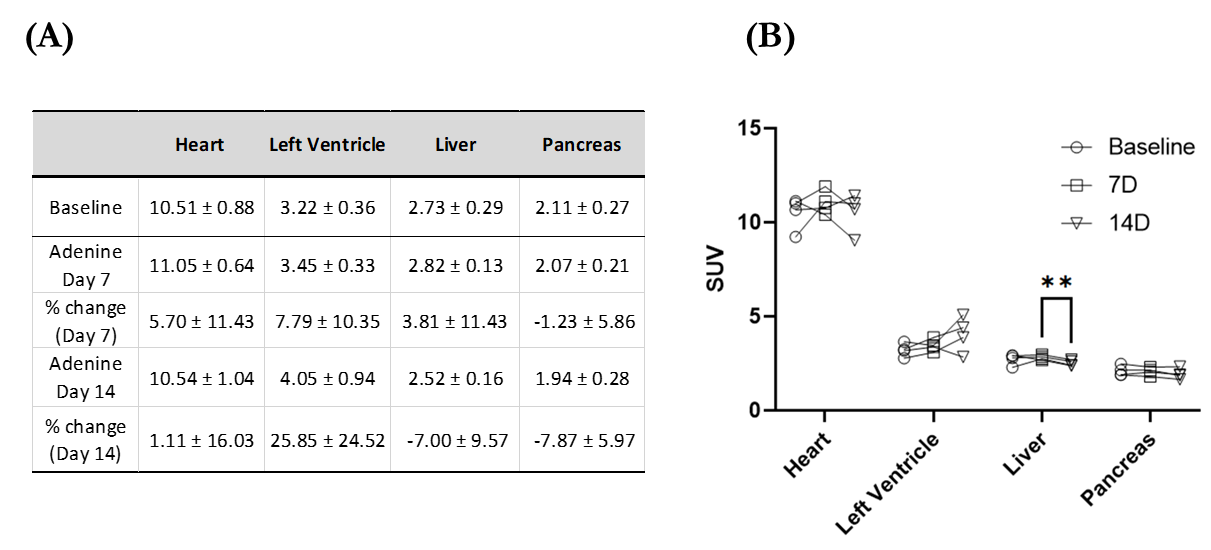
**

**Table S2.** Late (33 – 93 min) SUV values in the heart, left ventricle, liver, and pancreas following the intravenous injection of [^18^F]BCPP-BF. Average of late SUV values (n = 4) at baseline, Day 7, and Day 14. The data also include % change compared to baseline (A). Same SUV data as in (A) but displayed in a plot format (B). 2-way ANOVA, with Tukey's multiple comparison (**p=0.0013).

1. **In vitro autoradiography data with [^18^F]BCPP-BF**


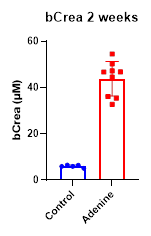


**Fig S3.** Measured creatine levels for mice used in the in vitro autoradiography study, n = 6 on normal (control) diet and n = 9 mice on adenine diet for two weeks.

**Fig S4.** [^18^F]BCPP-BF binding autoradiography in kidney tissue sections from Control mice and mice fed with adenine in their diet for 14 days (Adenine). Specific [^18^F]BCPP-BF Binding (mBq/mm2), calculated by subtracting the signal obtained co-incubation of the [^18^F]BCPP-BF with 10 µM BCPP-BF (left) and 10 µM Rotenone (right) from the Total signal. **** p<0.0001, two-tailed unpaired t-test.

1. **[^18^F]BCPP-BF semipreparative and analytical chromatograms**

**
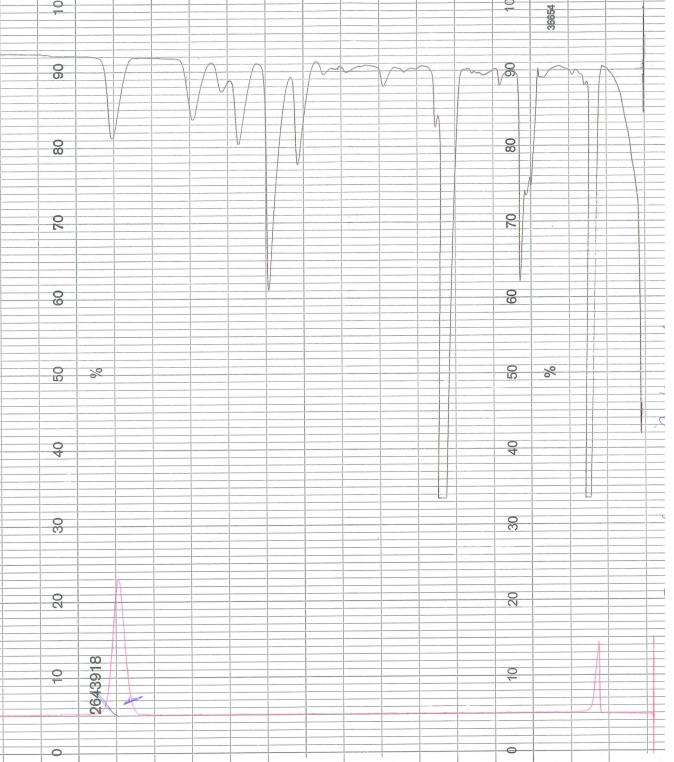
**

**Fig. S5.** Semipreparative HPLC chromatogram. Upper trace: Radioactivity trace. Lower trace: UV-trace. Each square corresponds to 1 minute.


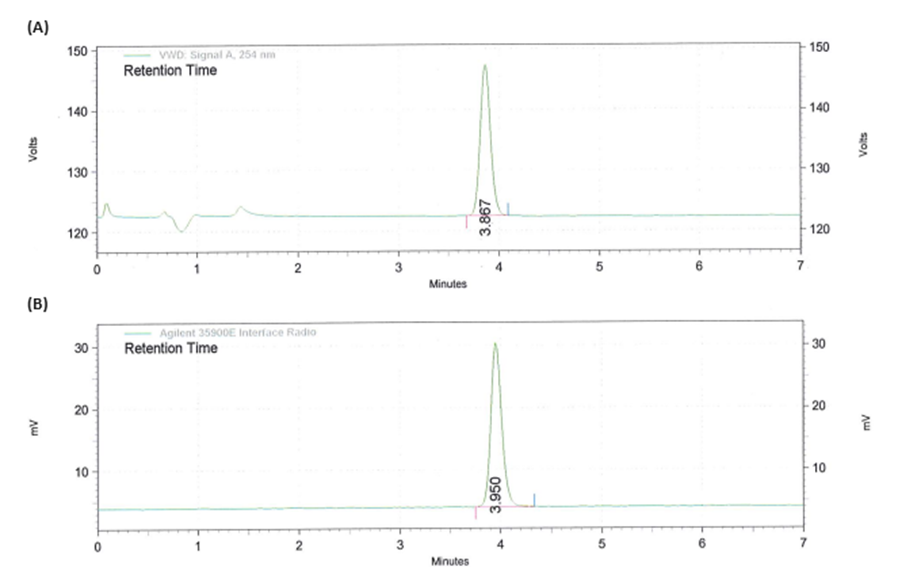


**Fig. S6.** HPLC chromatogram of [^18^F]BCPP-BF spiked with reference BCPP-BF. (A) UV-trace. (B) Radioactivity trace.
